# How do *Wolbachia* modify the *Drosophila* ovary? New evidences support the “titration-restitution” model for the mechanisms of *Wolbachia*-induced CI

**DOI:** 10.1186/s12864-019-5977-6

**Published:** 2019-07-24

**Authors:** Zhen He, Ya Zheng, Wen-Juan Yu, Yang Fang, Bin Mao, Yu-Feng Wang

**Affiliations:** School of Life Sciences, Hubei Key Laboratory of Genetic Regulation and Integrative Biology, Central China Normal University, Wuhan, 430079 People’s Republic of China

**Keywords:** *Wolbachia*, *Drosophila melanogaster*, Ovary, RNA-seq, miRNAs

## Abstract

**Background:**

Cytoplasmic incompatibility (CI) is the most common phenotype induced by endosymbiont *Wolbachia* and results in embryonic lethality when *Wolbachia*-modified sperm fertilize eggs without *Wolbachia*. However, eggs carrying the same strain of *Wolbachia* can rescue this embryonic death, thus producing viable *Wolbachia*-infected offspring. Hence *Wolbachia* can be transmitted mainly by hosts’ eggs. One of the models explaining CI is “titration-restitution”, which hypothesized that *Wolbachia* titrated-out some factors from the sperm and the *Wolbachia* in the egg would restitute the factors after fertilization. However, how infected eggs rescue CI and how hosts’ eggs ensure the proliferation and transmission of *Wolbachia* are not well understood.

**Results:**

By RNA-seq analyses, we first compared the transcription profiles of *Drosophila melanogaster* adult ovaries with and without the *w*Mel *Wolbachia* and identified 149 differentially expressed genes (DEGs), of which 116 genes were upregulated and 33 were downregulated by *Wolbachia* infection. To confirm the results obtained from RNA-seq and to screen genes potentially associated with reproduction, 15 DEGs were selected for quantitative RT-PCR (qRT-PCR). Thirteen genes showed the same changing trend as RNA-seq analyses. To test whether these genes are associated with CI, we also detected their expression levels in testes. Nine of them exhibited different changing trends in testes from those in ovaries. To investigate how these DEGs were regulated, sRNA sequencing was performed and identified seven microRNAs (miRNAs) that were all upregulated in fly ovaries by *Wolbachia* infection. Matching of miRNA and mRNA data showed that these seven miRNAs regulated 15 DEGs. *Wolbachia*-responsive genes in fly ovaries were involved in biological processes including metabolism, transportation, oxidation-reduction, immunity, and development.

**Conclusions:**

Comparisons of mRNA and miRNA data from fly ovaries revealed 149 mRNAs and seven miRNAs that exhibit significant changes in expression due to *Wolbachia* infection. Notably, most of the DEGs showed variation in opposite directions in ovaries versus testes in the presence of *Wolbachia*, which generally supports the “titration-restitution” model for CI. Furthermore, genes related to metabolism were upregulated, which may benefit maximum proliferation and transmission of *Wolbachia*. This provides new insights into the molecular mechanisms of *Wolbachia*-induced CI and *Wolbachia* dependence on host ovaries.

**Electronic supplementary material:**

The online version of this article (10.1186/s12864-019-5977-6) contains supplementary material, which is available to authorized users.

## Background

*Wolbachia* are widespread endosymbionts that frequently infect many insect species. They are well-known for their ability to manipulate host reproduction for their own propagation [1, 2]. Sperm-egg cytoplasmic incompatibility (CI) is the most common reproductive phenotype induced by *Wolbachia*, and it results in sterility or notably low hatch rates when mating occurs between *Wolbachia*-infected males and uninfected females. This indicates that infected males are “dead-end” hosts for *Wolbachia*; however, females carrying the same strain of *Wolbachia* can rescue zygotic lethality associated with CI [1, 3]. Thus, *Wolbachia*-mediated CI can be used as promising tools for the control of pests and disease vectors. Although this phenomenon has been known for around 45 years [4], the underlying molecular mechanisms of CI and how CI can be rescued are still not yet well understood and pose a great challenge to researchers who are working on it.

Cytological studies have shown that in CI embryos, paternal chromatin condensation and segregation defects during the first mitotic division following fertilization are the main causes of early embryonic mortality [5, 6]. Therefore, sperms from *Wolbachia*-infected males may be modified during spermatogenesis. Bourtzis et al. proposed a “modification/rescue” model to explain the production and rescue of CI, in which *Wolbachia* can modify male sperm during spermatogenesis, and females carrying the same *Wolbachia* can rescue such modification after insemination [7]. Thereafter several models were proposed to translate the “modification/rescue” concept [8, 9]. One of them is “titration-restitution” model, which proposed that during spermatogenesis, *Wolbachia* could titrate some essential components from the sperm, and the same strain of *Wolbachia* in the egg may compensate these critical factors, allowing embryogenesis to proceed normally [8]. According to this model, “modification” and “rescue” can be determined by the same gene(s) or by different genes. In the former case, the transition from titration to restitution after fertilization will be triggered by infected female hosts. In the latter case, one gene codes factors for titration and the other codes factors for restitution [8]. Some evidences support the former case. For instance, *Ance*, coding for angiotensin-converting enzyme, has been shown to be significantly upregulated in infected females but notably downregulated in infected males of *Drosophila simulans* [10]. Our previous work revealed that the expression level of *Hira*, coding for a negative regulator of histone gene expression, was higher in females, but lower in males when compared to uninfected *D. melanogaster* [11]. Recently, several studies have demonstrated that *Wolbachia* can secret gene products to host cells that may directly manipulate host reproduction [12–16]. For example, two adjacent genes, *cifA* and *cifB*, were identified from the *w*Mel strain to be responsible for the *w*Mel-induced sperm modification of CI, and *cifA* expression in ovaries can rescue CI in *D. melanogaster* [14, 16, 17]. Thus, Shropshire et al. established a “Two-by-One” model [17], which could partially support the latter case that shift from titration to restitution could be determined by different genes.

As *Wolbachia*-associated embryonic lethality is limited to paternal chromosomes, studies related to CI have mainly focused on males [11, 18–21]. For example, we previously found that the decreased *Hira* expression in male *D. melanogaster* was closely related to *Wolbachia*-induced CI [11]. Microarray analyses of the 3rd instar larval testes of *D. melanogaster* with and without *Wolbachia* revealed that among those DEGs, most genes putatively involved in reproduction exhibited downregulation in the presence of *Wolbachia* [18]. Comparative proteomics of the spermatheca and seminal receptacle (containing sperm proteins and seminal fluid proteins from their mates) from uninfected females mated with *Wolbachia*-infected or uninfected males identified many proteins including seminal fluid proteins that were downregulated due to *Wolbachia* infection [20]. Landmann et al. found that in CI embryos, a delay of histone H3.3 and H4 deposition occurred on the sperm nuclei during male pronuclei formation, which may cause the asynchronous development of male and female pronuclei and thus embryonic death [22]. However, there are few studies on *Wolbachia*-infected ovaries [23]. How *Wolbachia*-infected eggs can rescue CI, and how *Wolbachia* modify *Drosophila* ovary to benefit for their own survival and propagation are unclear.

Considering that *Wolbachia*-infected eggs can rescue embryonic lethality when fertilized with sperm produced by *Wolbachia*-infected males, but *Wolbachia*-uninfected eggs cannot, and that *Wolbachia* can be transferred to progeny mainly by the egg, we applied RNA-seq technology to compare the transcriptional profiles as well as sRNAs expression patterns between *Wolbachi*a-infected and uninfected adult ovaries of *D. melanogaster*. We identified 149 genes with at least a 2-fold change in expression (*p* < 5%). We also identified seven significantly differentially expressed miRNAs, which were all significantly upregulated in ovaries harboring *Wolbachia*. The target genes of differentially expressed miRNAs were further identified. The changed expression patterns of some genes caused by *Wolbachia* infection in ovaries and testes were compared. In general, our results, together with previous data, support the titration-restitution hypothesis for CI. These findings may provide new insights into *Wolbachia*/host interactions and especially the molecular mechanisms of CI induced by *Wolbachia*.

## Results

### Identification of mRNAs involved in *Wolbachia* infection in *Drosophila* ovaries

To investigate the rescue mechanisms involved in CI induced by *Wolbachia* and the impact of *Wolbachia* on maternal transmission, we first compared the mRNA profiles between *Wolbachia*-infected and uninfected *Drosophila* ovaries by RNA-seq. A total of 60,847,382 sequence reads for Dmel T (*Drosophila melanogaster* without *Wolbachia*) ovaries and 54,620,985 for Dmel *w*Mel (*D. melanogaster* infected *w*Mel *Wolbachia*) ovaries were generated. An overview of sequencing and assembly is outlined in Table 1. After removal of adaptor sequences, ambiguous reads and low-quality reads, 58,795,543 high-quality clean reads (96.6% of the raw data) of Dmel T remained, while 53,645,601 high-quality clean reads (98.2% of the raw data) of Dmel *w*Mel remained. Eighty-eight percent of clean reads had Phred-like quality scores at the Q30 level (an error probability of 0.001).

**Table 1:** Summary of RNA sequence assembly of *D. melanogaster* ovaries

Sample name	Raw reads	Clean reads	Clean bases	Error rate (%)	Q20 (%)	Q30(%)	GC content (%)
Dmel T	60,847,382	58,795,543	8.82G	0.03	95.48	88.09	52.98
Dmel *w*Mel	54,620,985	53,645,601	8.04G	0.03	95.4	88.22	52.61

Dmel T: *Drosophila melanogaster* treated with tetracycline, *Wolbachia*-free; Dmel *w*Mel: *Drosophila melanogaster* infected with *w*Mel *Wolbachia*

**Fig. 1:**
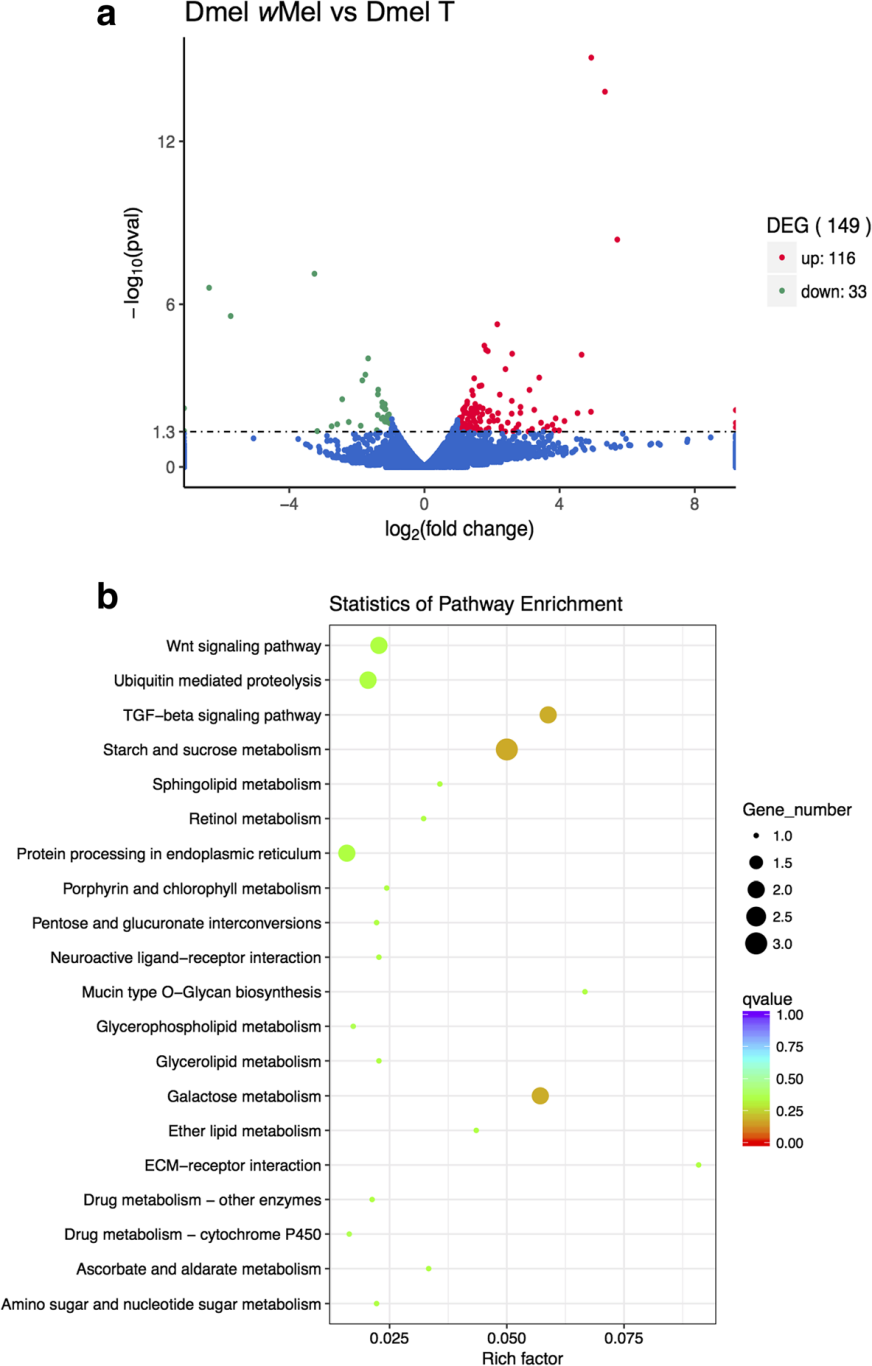
Volcanoplot (**a**) and KEGG pathway (**b**) of differentially expressed genes in the *Drosophila melanogaster* ovary due to the infection of *Wolbachia*. (**a**) Distribution of the relative expression of genes in the infected versus uninfected fly ovaries. Green dots represent downregulated genes; red dots represent upregulated genes and blue dots represent unchanged genes. (**b**) The 20 most enriched KEGG pathways based on the differentially expressed genes in the presence of *Wolbachia*. The x-axis shows the rich factor; the y-axis shows the pathway names. The size of each point represents the number of genes enriched in a particular pathway. The larger the rich factor and the smaller the *q*-value, the more significant the degree of enrichment. Dmel T: *Drosophila melanogaster* treated with tetracycline (without *Wolbachia*); Dmel *w*Mel: *Drosophila melanogaster* infected with *w*Mel *Wolbachia*

A total of 149 differentially expressed genes (DEGs) were identified, 116 of which were upregulated and 33 of which were downregulated in the presence of *Wolbachia* (Fig. 1a, Additional file 1). When these DEGs were subjected to KEGG (Kyoto Encyclopedia of Genes and Genomes) pathway analysis, we found that starch and sucrose metabolic pathways had the largest number of DEGs. Furthermore, the TGF-β signaling pathway, galactose metabolism, Wnt signaling pathway, ubiquitin-mediated proteolysis, and protein processing in endoplasmic reticulum were also enriched (Fig. 1b, Additional file 2). Gene Ontology (GO) annotation enrichment revealed that among the DEGs with known biological process, metabolism, organism process, cellular process, localization, transportation, and development were most prevalent (Additional file 3).

It is interesting that some genes encoding partners of cytochrome P450 (CYP) showed differential expression patterns. Four genes, including *Cyp4p1*, *Cyp12a5*, *Cyp6g1*, and *Cyp12a4*, were identified to be upregulated, while *Cyp6a13* was downregulated in the presence of *Wolbachia* (Additional file 1). These five genes belong to the CYP, E-class, group I according to FlyBase and are involved in oxidation-reduction processes (http://flybase.bio.indiana.edu).

### qRT-PCR validation of some DEGs induced by *Wolbachia* infection in ovaries and testes

**Fig. 2:**
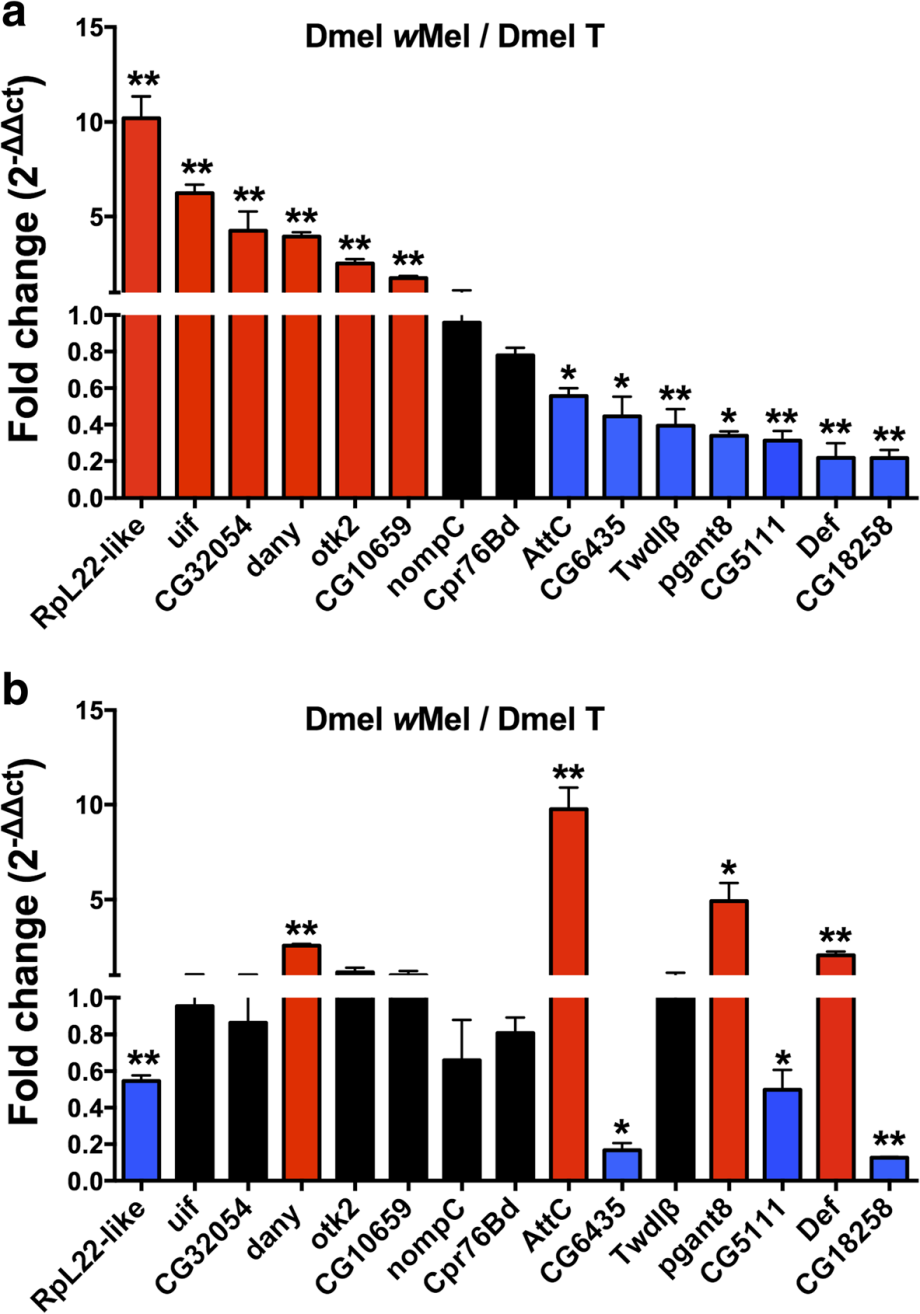
qRT-PCR validation of differentially expressed genes identified by RNA sequencing. a: gene expression in ovaries; b: gene expression in testes. A two-tailed Student’s *t*-test was performed to compare the significance between *Wolbachia*-infected (Dmel *w*Mel) and uninfected (Dmel T) fly ovaries (**a**) and testes (**b**) of candidate genes. Red color indicates upregulation; Blue color indicates downregulation; Black color indicates not significantly different.“/” represents the relative value; bars indicate standard error; “*” and “**” indicate significant differences with *p* < 0.05 and *p* < 0.01, respectively. *df* = 4, *n* = 3

In order to confirm the results observed during RNA-seq analysis, 15 differentially expressed genes based on large differences in expression in the RNA-seq analyses in addition to genes potentially associated with reproduction were selected for qRT-PCR to further investigate their expression profiles (Table 2). The results demonstrated that most of the genes (13) whose expression in ovaries measured by qRT-PCR exhibited similar changes as in the RNA-seq, with 6 genes (*RpL22-like*, *uif, CG32054, dany*, *otk2*, and *CG10659*) upregulated and 7 genes (*AttC*, *CG6435*, *Twdlβ*, *pgant8*, *CG5111*, *Def*, and *CG18258*) downregulated (Fig. 2a).

**Table 2:** Differentially expressed genes selected for qRT-PCR validation in adult ovaries in the presence of *Wolbachia*

Relative expression level	Gene symbol	Log_2_ Fold difference (Log_2_ Dmel *w*Mel/ Dmel T)	Biological functions
Up-regulated	*uif*	5.9542	Negative regulation of Notch signaling pathway
	*RpL22-like*	5.3421	Structural cosntituent of ribosome, protein metabolism
	*dany*	2.1552	Regulation of transcription from RNA polymerase II promoter involved in spermatogenesis
	*otk2*	1.0471	Imaginal disc-derived female genitalia morphogenesis
	*CG32054*	^a^No expression in Dmel T	Integral component of membrane
	*CG10659*	^a^No expression in Dmel T	Nitrogen compound metabolic process
Down-regulated	*CG5111*	−1.3823	Protein ubiquitination
	*Twdlβ*	−1.8867	Chitin-based cuticle development
	*Def*	−2.4344	Response to bacterium
	*AttC*	−2.5815	Response to bacterium
	*CG18258*	−3.2454	Lipid metabolic process
	*nompC*	−5.7343	Response to stimulus
	*Cpr76Bd*	−6.3724	Chitin-based cuticle development
	*CG6435*	^a^No expression in Dmel *w*Mel	Lysozyme activity
	*pgant8*	^a^No expression in Dmel *w*Mel	Oligosaccharide biosynthetic process

^a^No expression in Dmel T or Dmel *w*Mel indicate that the gene can not be detected by RNA-seq

Considering that young *Wolbachia*-infected male flies can induce high level of CI [11, 24], to test whether the gene expression levels change due to *Wolbachia* infection is in a reverse trend in testes, we also detected the expression level of these genes in 1-day-old fly testes. Four genes (*dany*, *AttC*, *pgant8*, and *Def*) were upregulated and four genes (*RpL22-like*, *CG6435*, *CG5111*, and *CG18258*) were downregulated in the presence of *Wolbachia* (Fig. 2b). Of these selected genes, *RpL22-like* showed the highest upregulation in *Wolbachia*-infected ovaries (around 10 times) and downregulation in *Wolbachia*-infected testes, which were similar to the expression patterns of *Ance* and *Hira* [10, 11]. Other genes of interest including *AttC*, *pgant8*, and *Def* were downregulated in *Wolbachia*-infected ovaries but upregulated in infected testes.

### sRNA annotations in Dmel *w*Mel and Dmel T ovaries

**Fig. 3:**
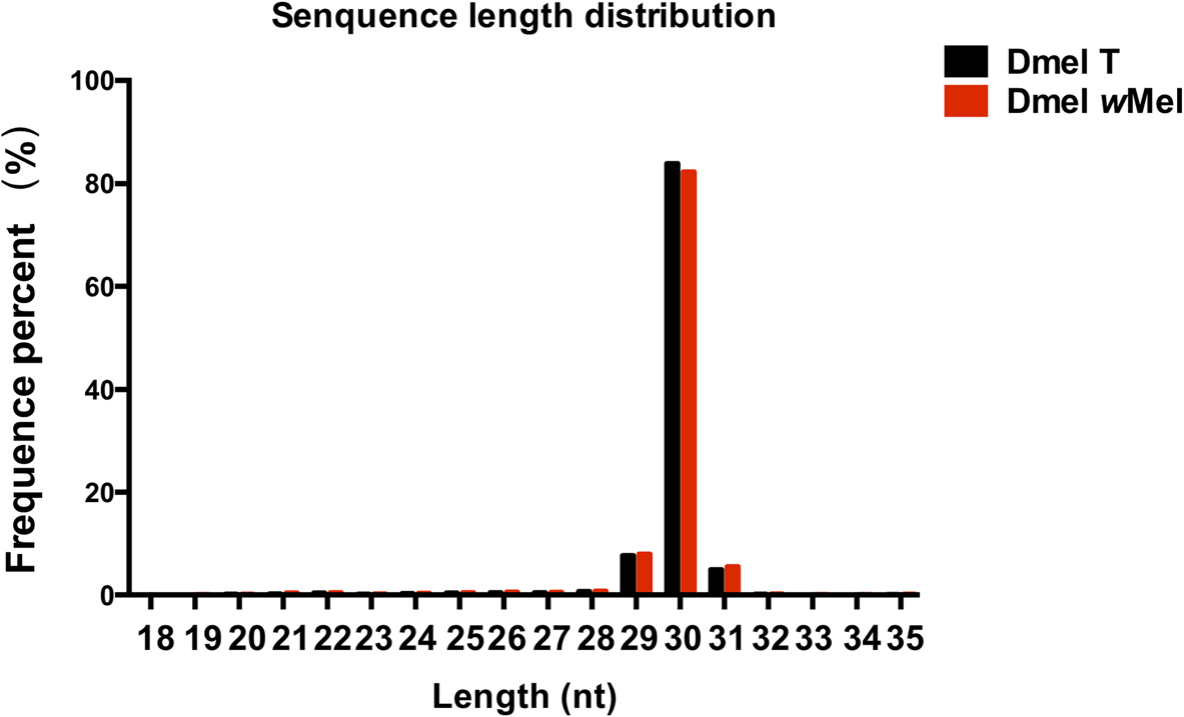
The length of distribution of small RNAs (sRNAs) in Dmel T and Dmel *w*Mel ovaries. Dmel T: *Drosophila melanogaster* treated with tetracycline (without *Wolbachia*); Dmel *w*Mel: *D. melanogaster* infected with *w*Mel *Wolbachia.* nt: nucleotides

**Fig. 4:**
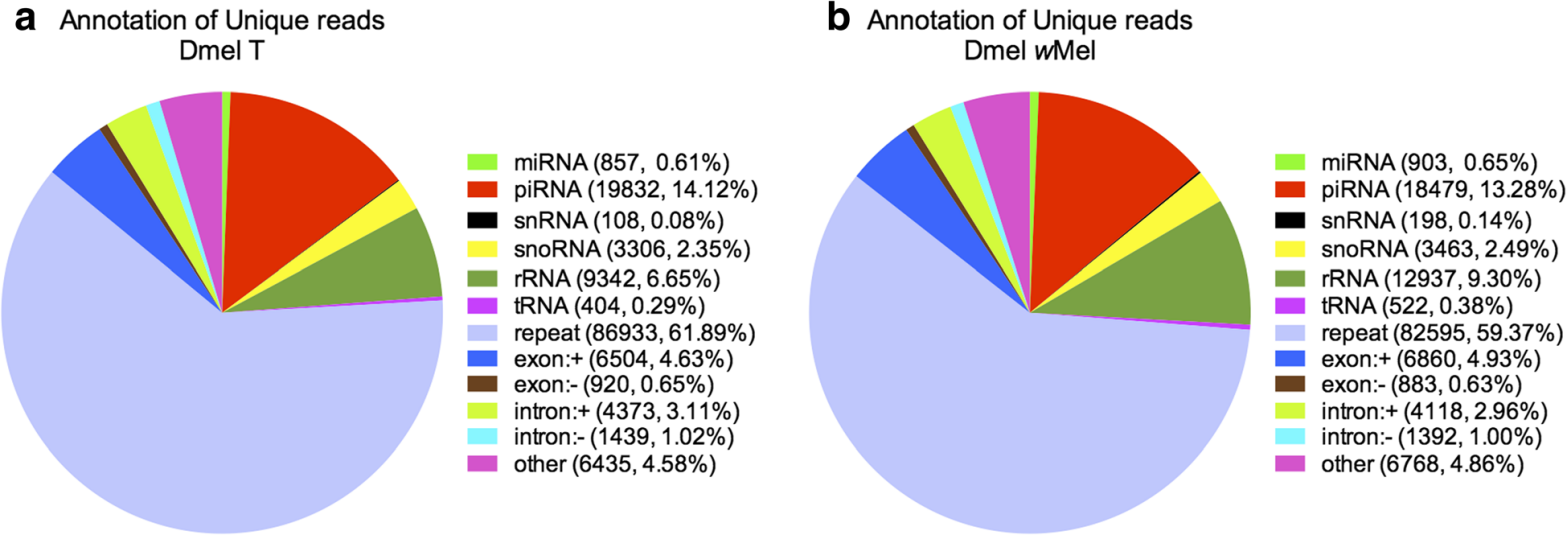
Classification of sRNAs in Dmel T and Dmel *w*Mel ovaries. Dmel T: *Drosophila melanogaster* treated with tetracycline (without *Wolbachia*); Dmel *w*Mel: *D. melanogaster* infected with *w*Mel *Wolbachia*. miRNA: microRNA; piRNA: Piwi-interacting RNA; snRNA: small nuclear RNA; sonRNA: small nucleolar RNA; rRNA: ribosome RNA; tRNA: transfer RNA; repeat: repeative sRNA sequences; exon:^+^: exon sense; exon:^-^: exon antisense; intron:+: intron sense; intron:^-^: intron antisense; other: unannotated sRNA

An increasing body of evidence suggests that non-coding RNAs, including small RNAs (sRNAs), play a critical role in regulating gene expression and thus in reproduction and development [25, 26]. To study the responses of sRNAs in ovaries to *Wolbachia* infection, two sRNA libraries were constructed from Dmel T and Dmel *w*Mel virgin fly ovaries with an average of 8.11 million raw clean reads, ranging from 18 nt to 35 nt in length. The peak size was 30 nt, followed by 29 nt and 31 nt (Fig. 3), indicating that PIWI-interacting RNAs (piRNAs) might be the major component of ovary sRNAs. An overview of sequencing and assembly is outlined in Table 3. A total of 7,700,845 high-quality clean reads of Dmel T remained; 7,690,372 high-quality clean reads of Dmel *w*Mel remained. Nighty-6 % of clean read data had Phred-like quality scores at the Q30 level. The sRNAs were divided into twelve classes, such as miRNAs, piRNAs and rRNAs (Fig. 4). The percentage of piRNAs in unique reads in Dmel T and Dmel *w*Mel were highest: 14.12% (including 11.54% of novel piRNAs) and 13.29% (including 10.77% of novel piRNAs), respectively. This is consistent with Fig. 3 where piRNAs were the major component of sRNAs in the ovary. Furthermore, the percentage of known miRNAs in unique reads in Dmel T and Dmel *w*Mel were 0.61 and 0.65%, and rRNA accounted for 6.65 and 9.30% in Dmel T and Dmel *w*Mel, respectively (Fig. 4a and b).

**Table 3:** Basic characteristics of tags in sRNA libraries from *D. melanogaster* ovaries

Sample name	Raw reads	Clean reads	Unique reads	Q20(%)	Q30(%)	GC content (%)
Dmel T	8,109,689	7,700,845	153,804	98.04	96.3	47.51
Dmel *w*Mel	8,116,530	7,690,372	160,901	97.94	96.12	47.47

Dmel T: *Drosophila melanogaster* treated with tetracycline, *Wolbachia*-free; Dmel *w*Mel: *Drosophila melanogaster* infected with *w*Mel *Wolbachia*

### Identification of differentially expressed miRNAs involved in *Drosophila* ovaries and testes

**Fig. 5:**
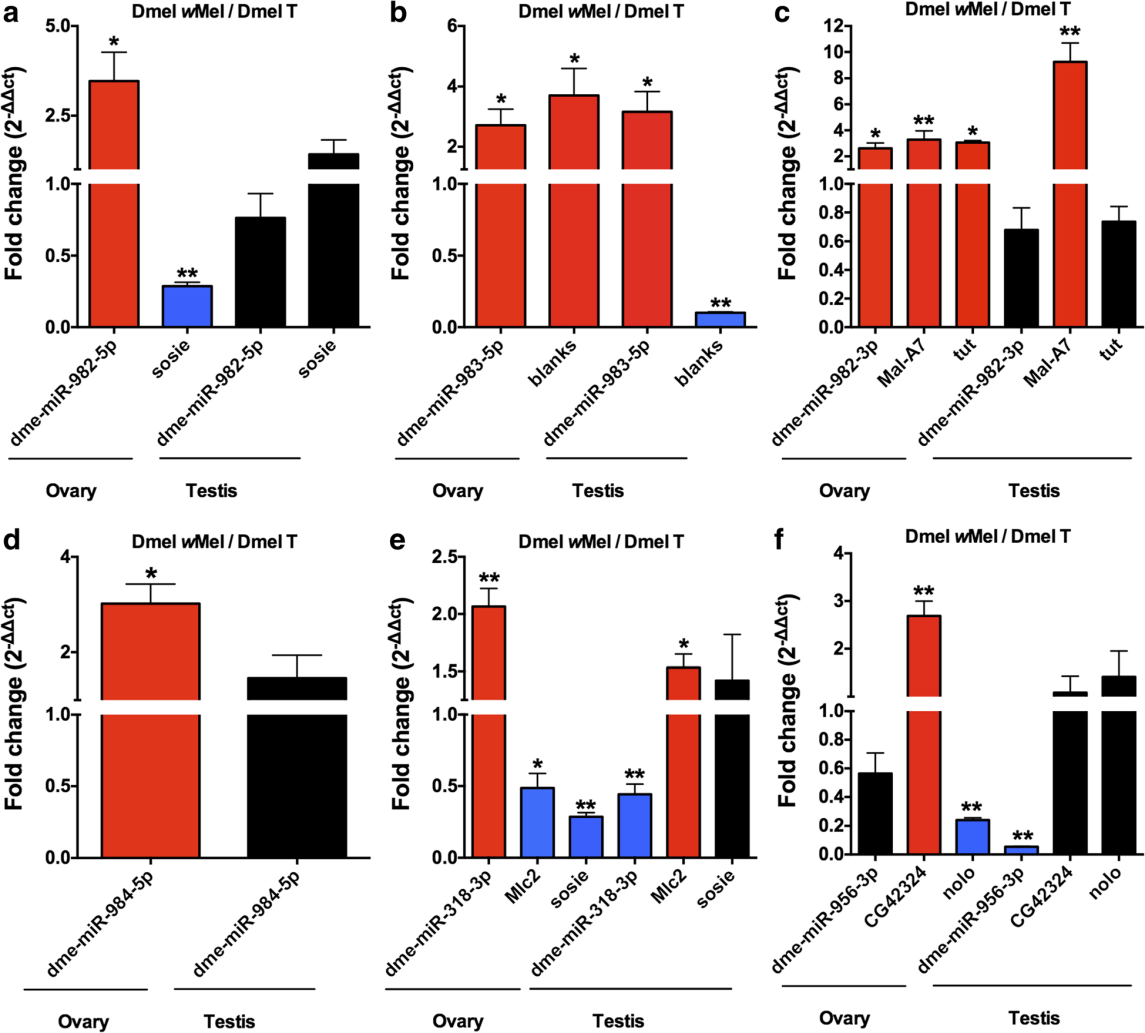
qRT-PCR confirmation of differentially expressed miRNAs and their targets in adult gonads of *Drosophila melanogaster*. Dmel T: *D. melanogaster* treated with tetracycline (without *Wolbachia*); Dmel *w*Mel: *D. melanogaster* infected with *w*Mel *Wolbachia*. Red color indicates upregulation; Blue color indicates downregulation; Black color indicates not significantly different. A two-tailed Student’s *t*-test was performed to compare the significance between *Wolbachia*-infected and uninfected fly ovaries and testes of candidate miRNAs and their target genes. “/” represents the relative value; Bars indicate standard error, “*” and “**” indicate significant differences with *p* < 0.05 and *p* < 0.01, respectively. *df* = 4, *n* = 3

In *Wolbachia*-infected female ovaries, seven miRNAs were identified as upregulated compared with uninfected controls (Table 4). However, no significantly downregulated miRNAs were observed. To verify the results of sRNA-seq, the expression profiles of these seven miRNAs were further measured by qRT-PCR. Five miRNAs (dme-miR-982-5p, dme-miR-983-5p, dme-miR-982-3p, miR-984-5p, and dme-miR-318-3p) exhibited significantly upregulated in the presence of *Wolbachia* (Fig. 5), which is in conformity with sRNA-seq analyses. However, dme-miR-956-3p showed no significant change. We could not detect dme-miR-983-3p expression in ovaries by qRT-PCR. As for the testes, dme-miR-983-5p manifested notable upregulation, whereas dme-miR-318-3p and dme-miR-956-3p showed significant downregulation due to *Wolbachia* infection (Fig. 5b, e, f). However, dme-miR-982-5p, dme-miR-982-3p, and miR-984-5p did not show a significant difference between *Wolbachia*-infected and uninfected fly testes (Fig. 5a, c and d). Again, we did not detect the expression level of dme-miR-983-3p in testes (data not shown).

**Table 4:** Identification of differentially expressed miRNAs and their targets in adult ovaries of *Wolbachia*-infected flies relative to uninfected ones

miRNA	Log_2_ Fold difference (Log_2_ Dmel *w*Mel/ Dmel T)	Target gene	Fold difference (Log_2_ Dmel *w*Mel/ Dmel T)	Biological functions of target genes
dme-miR-982-5p	3.9511	*sosie*	−1.1911	Ovarian follicle cell migration
		*CG9411*	2.7156	−//−
dme-miR-983-5p	2.8377	*blanks*	2.0137	Regulation of chromatin silencing
dme-miR-982-3p	1.8142	*nompC*	−5.7343	Cellular response to mechanical stimulus
		*Mal-A7*	3.5326	Carbohydrate metabolic process
		*tut*	1.4346	Negative regulation of translation
dme-miR-984-5p	1.7784	*–*		
dme-miR-318-3p	1.3118	*Mlc2*	−1.1691	Muscle system process, myofibril assembly
		*Oamb*	1.0821	Ovulation, male courtship behavior
		*sosie*	−1.1911	Ovarian follicle cell migration
dme-miR-956-3p	1.2088	*tipE*	1.711	Regulation of sodium ion transport
		*nolo*	−1.7481	Ventral cord development
		*CG42324*	1.0045	−//−
dme-miR-983-3p	1.1192	*scro*	1.5012	Dendrite morphogenesis
		*Rab3-GEF*	1.1454	Regulation of cell cycle
		*Neurochondrin*	−1.0955	Muscle system process
		*CG32816*	1.4094	−//−

### Target gene prediction of differentially expressed miRNAs

**Fig. 6:**
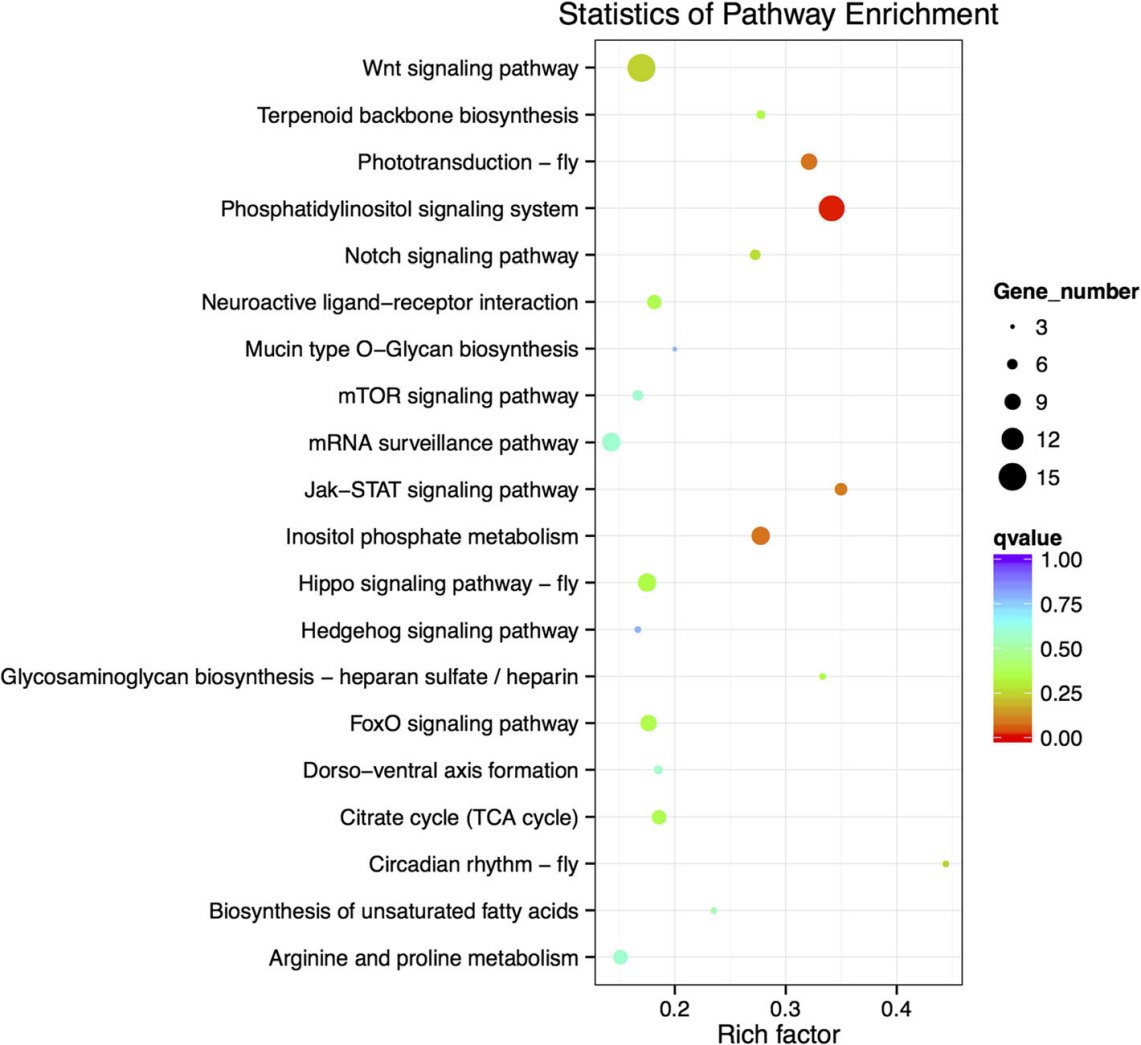
The 20 most enriched KEGG pathways based on target genes of differentially expressed miRNAs in *Wolbachia*-infected and uninfected adult ovaries. The x-axis is the rich factor, and the y-axis is the pathway terms. The size of each point represents the number of genes enriched in a particular pathway. The larger the rich factor and the smaller the *q*-value, the more significant the degree of enrichment

To further determine the biological functions of the differentially expressed miRNAs, miRanda was used to predict the target genes of these seven miRNAs. When target genes of differentially expressed miRNAs were further subjected to KEGG pathway analysis, the 20 most enriched pathways included phosphatidylinositol signaling, inositol phosphate metabolism, the Wnt signaling pathway, and the JAK-STAT signaling pathway (Fig. 6, Additional file 4). The GO annotation enrichment displayed that metabolic process, development process, and response to stimulus were included in the biological processes (Additional file 5).

### Association analysis of *Wolbachia* infection-related miRNAs and mRNAs

In general, miRNAs have multiple target genes. The differentially expressed target genes that may be regulated by these seven *Wolbachia*-responsive miRNAs are presented in Table 4. qRT-PCR was performed to further examine how these target genes are associated with their *Wolbachia*-responsive miRNAs in ovaries and testes and thus with CI. The miRNAs dme-miR-982-5p and dme-miR-318-3p targeted the same gene, *sosie*, which is involved in ovarian follicle cell migration [27]. qRT-PCR results were consistent with RNA-seq analyses that *sosie* was significantly downregulated in fly ovaries due to *Wolbachia* infection, indicating that *sosie* is negatively regulated by these two miRNAs (Fig. 5a and e). The gene *blanks*, required for postmeiotic spermiogenesis [28], was positively regulated by dme-miR-983-5p in ovaries and negatively regulated in testes (Fig. 5b). The miRNA dme-miR-982-3p targeted three genes: *nompC*, *Mal-A7*, and *tut*. As shown in Fig. 2, *nompC* did not show a significant difference in expression between *Wolbachia*-infected and uninfected fly ovaries and testes. In contrast, *Mal-A7* and *tut* were confirmed by qRT-PCR to be postively regulated by dme-miR-982-3p in fly ovaries (Fig. 5c). *Mal-A7* was also significantly upregulated in *Wolbachia*-infected testes, though there was no difference in the expression of this miRNA in testes (Fig. 5c). The miRNA dme-miR-318-3p was significantly upregulated in ovaries but downregulated in testes due to *Wolbachia* infection. Its target *Mlc2* was negatively regulated by dme-miR-318-3p in both ovaries and testes, exhibited downregulation in ovaries but upregulation in testes when compared to paired gonads without *Wolbachia* (Fig. 5e). In addition to *Mlc2* and *sosie*, the third target of dme-miR-318-3p is *Oamb*, which showed no difference in expression in both ovaries and testes (data not shown). The expression of dme-miR-956-3p was not significantly changed by *Wolbachia* infection in ovaries, while its targets *CG42324* and *nolo* were upregulated and downregulated, respectively, consistent with our RNA-seq data (Fig. 5f, Additional file 1). The third target gene of dme-miR-956-3p is *tipE*, which did not exhibit significant differences in both ovaries and testes in the presence and absence of *Wolbachia* (data not shown).

## Discussion

For years, the molecular mechanisms of CI induced by *Wolbachia* remained unclear. Previous studies focusing on *Wolbachia*-infected males revealed a series of data on host gene expression changes, which may help to elucidate the possible molecular mechanisms of CI [11, 18–21]. Nevertheless, how *Wolbachia*-infected eggs may rescue the CI defect caused by *Wolbachia*-modified sperm and nullify embryonic death, and what makes the ovary a suitable place for *Wolbachia* to survive and transmit have not yet been elucidated. Therefore, we compared the transcriptional profiles including sRNAs between *Wolbachia*-infected and uninfected ovaries of *D. melanogaster*.

By using RNA-seq, we identified 149 differentially expressed genes in fly ovaries due to *Wolbachia* infection, of which 77.85% (116) showed significant upregulation. This generally altered gene expression pattern caused by *Wolbachia* infection is the reverse of that found in our previous work on the male aspect [20], where we identified 83 differentially expressed proteins by comparative proteomics in the spermatheca and seminal receptacle from uninfected females mated with *Wolbachia*-infected or uninfected males, and 71.08% (59) of them showed downregulation in the presence of *Wolbachia* [20]. Our findings broadly support the titration-restitution model proposed by Poinsot et al. to explain “modify/rescue” mechanism of CI. According to this model, *Wolbachia* may titrate-out some essential molecules from the sperm, and the same strain of *Wolbachia* in the egg can restore these critical factors, allowing embryos to develop normally [8].

Some studies have demonstrated that some genes potentially related to CI showed the opposite expression pattern in *Wolbachia*-infected male and female hosts. For instance, *Ance* and *Hira* were both upregulated in infected females and downregulated in infected males of *Drosophila*, which might be linked to CI expression [10, 11]. In the present study, we found a number of DEGs that were associated with cellular processes by influencing the expression of genes involved in transcription and translation. For example, *RpL22-like* (coding for ribosome protein L22e), *Ptx1* (coding for paired-type homeobox protein), *blanks* (coding for siRNA binding protein and associated with regulation of chromatin silencing), and *scro* (coding for one of the homeobox proteins) were all upregulated in the presence of *Wolbachia*. This is consistent with the case of *Encarsia suzannae* infection with *Cardinium* strain *c*Eper1, which also can induce CI in its insect hosts, where the authors found upregulation of several female-biased genes encoding ribosomal proteins [29], suggesting increased general translational activity in female hosts. Notably, *RpL22-like* and *blanks* were significantly downregulated in the testes of *D. melanogaster* due to *Wolbachia* infection (Fig. 2b and 5b). This altered expression pattern is consistent with the work with *Ance* [10] and *Hira* [11] and also matches the “titration-restitution” model for CI [8].

In this study, we also identified multiple differentially expressed genes involved in immunity in ovaries between *Wolbachia*-infected and uninfected *D. melanogaster*, most of which were downregulated including *AttC* (coding for Attacin-C) and *Def* (coding for Defensin). The altered expression of these two genes caused by *Wolbachia* infection was further confirmed by qRT-PCR in both ovaries and testes. The expression levels of both *AttC* and *Def* were significantly decreased in ovaries but increased in testes. The decreased expression of immune-related genes in ovaries may reflect a reduced pathogenicity caused by the symbiont and an increased tolerance of the host to *Wolbachia*, thus offering the benefit of maximum proliferation and transmission of the bacteria. On the other hand, the reduced immune response may also allow the infected eggs to fit the modified sperm once fertilization occurs, so as to ensure normal embryogenesis. This altered expression pattern is generally opposed to that in larval testes [18]. By microarray analyses, we previously revealed that most of the immune-associated genes were upregulated in the 3rd instar larval testes in the presence of *Wolbachia* [18]. Again, the opposite expression pattern between ovaries and testes of these immune genes is also in accord with the “titration-restitution” model [8].

Previous work has demonstrated that miRNAs could function in *Wolbachia*/host interactions [30–35]. For instance, in *Aedes aegypti*, the *w*Melpop-CLA strain of *Wolbachia* can alter aae-miR-2940 to increase metalloprotease gene expression for self-maintenance in mosquito hosts [30]. In insects, many lines of evidence revealed that miRNAs could operate in ovarian development [36–40]. Here, we reported for the first time that *Wolbachia* could modulate miRNAs of host ovaries. Upregulation of both dme-miR-318-3p and dme-miR-982-5p may ensure downregulation *sosie* in response to *Wolbachia* infection. The involvement of *sosie* in the coordination of migrations of ovarian border cells and outer follicle cells during mid-oogenesis has been demonstrated [27]. Interestingly, a recent work discovered a novel *Wolbachia* tropism (the polar cells) in *Drosophila* ovary, which may facilitate efficient vertical transmission of the endosymbiont [41]. During stage 9 of oogenesis, the anterior polar cells, along with a few surrounding border cells, invasively migrate through the nurse cells and towards the oocyte. This migration provides an adequate opportunity for *Wolbachia* to traverse from polar cells to the germline [41]. Downregulation of *sosie* may slow the migration of ovarian border cells and outer follicle cells thus aid *Wolbachia* in traversing into the germ line during oogenesis of *Drosophila*. Moreover, the opposite expression pattern of dme-miR-318-3p in ovaries (upregulated) and testes (downregulated) caused by *Wolbachia* infection is in accordance with its target *Mlc2* expression pattern. *Mlc2* codes for myosin light chain 2, which is normally involved in myofibril assembly. Recently, Zhang et al. found *Mlc2* could also act as a transcription factor that can regulate the expression of nicotinamide adenine dinucleotide phosphate oxidase 2 to enhance oxidative stress in a phosphorylation-dependent manner [42]. The consistently altered expression patterns of dme-miR-318-3p and *Mlc2* in response to *Wolbachia* suggest that miRNA pathways are involved in the induction and rescue of CI. And once again, the reverse altered expression patterns of dme-miR-318-3p and its target *Mlc2* in ovaries and testes due to *Wolbachia* infection are consistent with the “titration-restitution” hypothesis [8].

Among those significantly upregulated genes, a large number are involved in metabolism processes and transport. These are consistent with findings in the previous experimental literature [18, 43, 44]. Caragata et al. found that numbers of genes encoding digestive enzymes, such as serine proteases or trypsin, showed increased expression in female *Aedes fluviatilis* in the presence of native *w*Flu *Wolbachia* [45]. In this research, we found that *CG10659* (coding Acyl-CoA N-acyltransferase) displayed upregulation in the ovary. However, it did not show significant change in expression in the testis in response to *Wolbachia* infection (Fig. 2). This indicates that the increase in *CG10659* expression resulting from *Wolbachia* infection is ovary-specific. *CG32054*, coding for a general substrate transporter, was also upregulated in *Wolbachia*-infected *Drosophila* ovaries but did not significantly alter due to *Wolbachia* infection in testes. These are consistent with our previous microarray analyses of the transcriptional profiles of *D. melanogaster* larval testes with and without *w*Mel *Wolbachia* [18], suggesting that there might be some materials specially required in host ovaries for bacterial survival and propagation. This is not in accord with titration-restitution hypothesis. Instead, it may reflect a special requirement for *Wolbachia* propagation through the hosts’ eggs. The *Drosophila* ovary proteome and the female mosquito transcriptome also showed widespread upregulation of gene products involved in the transport and metabolism of amino acid, carbohydrate and lipid transporters [23, 45]. The genome sequence of the *w*Mel strain showed that *Wolbachia* do not contain the complete set of metabolic pathways present in free-living bacteria [46]. Therefore, *Wolbachia* may not only upregulate the metabolism of their hosts for cannibalization of host resources, but also depend on a large assortment of transporters to obtain sufficient energy and critical components to promote their own proliferation and propagation [45–47].

## Conclusions

In this study, mRNAs and miRNAs in fly ovaries with and without *Wolbachia* were compared. Our analyses revealed 149 mRNAs and seven miRNAs that exhibit significant changes in expression due to *Wolbachia* infection. These genes are involved in metabolism, transportation, oxidation-reduction, development, and other functions, suggesting a wide effect of *Wolbachia* on their female hosts. The differentially expressed genes are generally varied in opposite directions in male versus female flies in the presence of *Wolbachia*, which strongly supports the “titration-restitution” model for explaining CI mechanism. Furthermore, genes related to oxidation and reduction, as well as immunity, are also altered in fly ovaries by *Wolbachia* infection, reflecting a protection for host ovaries, thus ensuring the maximum proliferation and propagation of the bacteria. Our data provide insights into mechanisms of *Wolbachia*/host interactions, especially the *Wolbachia*-induced CI and *Wolbachia* dependence on host ovaries.

## Methods

### Fly stocks and rearing

*Drosophila melanogaster* flies were reared on a standard cornmeal- and molasses-based diet. Stocks were maintained at a constant temperature of 25 °C and under non-crowded conditions (approximate 200 eggs per 50-ml vial of media in 150-ml conical flasks) [48]. All flies were kept on a 12-h light/dark cycle. The *D. melanogaster* infected with *w*Mel *Wolbachia*, referred to as Dmel *w*Mel, was a gift from Professor Scott O’Neill at Monash University, Australia. *Wolbachia*-uninfected lines from Dmel *w*Mel, referred to as Dmel T, were subsequently generated through tetracycline treatment as previously described [49] and verified to be *Wolbachia*-free by PCR using *Wolbachia* surface protein (*wsp*) primers (Additional file 6). The cured flies were reared on normal tetracycline-free medium for at least six generations to eliminate any influences of residual tetracycline before they were used in experiments [50].

### RNA preparation

Eighty pairs of ovaries for each replicate were dissected from 4-day-old virgin females of Dmel T and Dmel *w*Mel flies, respectively. RNA preparation and sequencing were performed by Novogene Bioinformatics Technology Co., Ltd. (Beijing, China). Briefly, total RNA was extracted using TRIzol reagent following the manufacturer’s instructions. Total RNA integrity was determined using the RNA Nano 6000 Assay Kit of the Bioanalyzer 2100 system (Agilent Technologies, CA, USA), and concentration was measured using the Qubit RNA Assay Kit in the Qubit 2.0 Fluorometer (Life Technologies, CA, USA).

### RNA sequencing

Sequencing libraries were prepared using NEBNext® Ultra™ RNA Library Prep Kit for Illumina (NEB, USA) according to the manufacturer’s recommendations. Briefly, mRNA was isolated from total RNA using Oligo-dT beads and then fragmentation was performed using NEBNext First Strand Synthesis Reaction Buffer (5X). First-strand cDNA was synthesized with random hexamer primers and M-MuLV Reverse Transcriptase. Second-strand cDNA was subsequently synthesized with DNA polymerase I and RNase H. Double-stranded cDNA fragments were purified with the AMPure XP system (Beckman Coulter, Beverly, USA) and adenylated at the 3′ ends. The NEBNext Adaptor with a hairpin loop structure was ligated to prepare for hybridization. The adaptor-ligated cDNA was used as a template to carry out PCR with Phusion High-Fidelity DNA polymerase and Universal PCR primers. Then the PCR products were purified with the AMPure XP system, and library quality was determined on the Agilent Bioanalyzer 2100 system. Finally, the cDNA library preparations were sequenced on an Illumina HiSeq 4000 platform and 150 bp paired-end reads were produced. Clean reads were obtained by removing reads containing adapter, ambiguous reads (the ratio of reads with poly N was greater than 10%, N indicated that base cannot be determined) and low-quality reads (The number of bases whose mass < =20 accounted for more than 50% of the total read length).

### Differential gene expression calculation and pathway analysis

Differentially expressed genes were selected based on a fold change > = 2 and a *p* value < 0.05 with three biological replicates. KEGG pathway (Kyoto Encyclopedia of Genes and Genomes) enrichment analysis of DEGs was performed using KOBAS [51]. Gene Ontology (GO) enrichment analysis of differentially expressed genes was performed by using the GOseq R package.

### Small RNA library construction for Illumina sequencing

Sequencing libraries were carried out using NEBNext Multiplex Small RNA Library Prep Set for Illumina (NEB, USA) following the manufacturer’s protocols. Briefly, the NEB 3′ adaptor was ligated to the 5′ and 3′ ends of miRNA using T4 ligase. The first-strand cDNA was synthesized using M-MLV Reverse Transcriptase. PCR amplification was conducted using LongAmp Taq 2X Master Mix. The PCR products were purified and then assessed on the Agilent Bioanalyzer 2100 system. At last, the cDNA library constructs were sequenced on an Illumina HiSeq 2500 platform and 50-bp single-end reads were generated. Clean reads were obtained by removing reads with 5′ primer contaminants, without 3′ primers or the insert tag, ambiguous reads (the ratio of reads with poly N was greater than 10%, N indicated that base cannot be determined), poly A/T/G/C reads and low-quality reads (The number of bases whose mass < =5 accounted for more than 50% of the total read length). Differentially expressed miRNAs were selected based on a fold change > = 2 and a *p* value < 0.05 with three biological replicates.

### Target gene prediction and functional enrichment

Candidate targets of differentially expressed miRNAs were predicted by miRanda [52]. GO enrichment analysis was used to target gene candidates of differentially expressed miRNAs. KOBAS [51] was used to test the statistical enrichment of the target gene candidates in the KEGG pathways.

### Quantitative reverse transcriptase PCR (qRT-PCR)

Forty pairs of ovaries for each biological replicate were dissected from 4-day-old virgin females of Dmel T and Dmel *w*Mel flies, respectively. Sixty pairs of testes for each biological replicate were dissected from 1-day-old males of Dmel T and Dmel *w*Mel flies, respectively. The qRT-PCR experiments were conducted using a CFX connect™ real-time system (BioRad). Total RNA was extracted using TRIzol (Invitrogen). DNA contamination was digested with RNase-free DNase I (Takara). The first-strand cDNA was synthesized from about 2 μg of total RNA using M-MLV reverse transcriptase (Invitrogen) and Oligo dT_18_ primer (Takara) at 37 °C for 50 min. Specific primers were designed based on sequences from the FlyBase database (Additional file 6). qPCR was performed with a Platinum SYBR Green qPCR superMix (Takara). Each reaction mixture contained 10 μl of 2× SYBR premix, 0.3 μl of forward and reverse primer (10 μM), respectively, 2 μl of cDNA template diluted 5-fold with deionized H_2_O, and deionized H_2_O to a final volume of 20 μl. The cycling program was 95 °C for 2 min, followed by 40 cycles of 95 °C for 10 s, 56–58 °C (based on various primers) for 20 s and 72 °C for 20 s, and then a melting curve was constructed from 55 °C to 98 °C. The relative expression of each gene was calibrated against the reference gene (*rp49*) using 2^-ΔCT^ (ΔC_T_ = C_T, target gene_ - C_T, rp49_). Three biological replicates and two technical replicates for each biological replicate were performed.

### miRNA expression patterns

The experiments were performed on a CFX connect™ real-time system (BioRad) with three biological replicates and two technical replicates. Forty pairs of 4-day-old virgin female ovaries and sixty pairs of 1-day-old male testes were dissected for each biological replicate. Total RNA was extracted with a miRNeasy extraction kit (TIANGEN catalog no. DP501) together with an RNase-Free DNase set. RNA quality was estimated with a Nanodrop spectrophotometer and by agarose gel electrophoresis. The expression of miRNA was checked by using the SYBR Prime-Script miRNA RT-PCR Kit (TIANGEN catalog no. FP401). Total RNA (2 μg) was reverse transcribed, and 30 ng cDNA products were added to a 20-μL quantification system. The primers used in this study are shown in Additional file 6. The reactions were incubated at 95 °C for 30 s, followed by 40 cycles of 95 °C for 5 s and 65 °C for 34 s. A dissociation curve was obtained to ensure that only one product was amplified after the amplification phase. The relative expression of each miRNA was normalized against the reference gene (U6 snRNA) using 2^-ΔCT^ (ΔC_T_ = C_T, target miRNA_ - C_T, U6 snRNA_).

### Statistical analyses

For all experiments, a two-tailed Student’s *t*-test was performed. All data were represented as the means ± standard error (SE, n = 3) and were graphed by GraphPad Prism 6.

## Additional files

Additional file 1: Differentially expressed genes in ovaries of *D. melanogaster* induced by *Wolbachia* infection. (XLS 57 kb)

Additional file 2: The 20 most enriched KEGG pathways from enrichment analysis of differentially expressed genes in ovaries of *D. melanogaster* induced by *Wolbachia* infection. (XLS 30 kb)

Additional file 3: GO analysis of differentially expressed genes in ovaries of *D. melanogaster* induced by *Wolbachia* infection*. (XLS 53 kb)*

Additional file 4: The 20 most enriched KEGG pathways based on target genes of differentially expressed miRNAs in ovaries of *D. melanogaster* induced by *Wolbachia* infection. (XLS 33 kb)

Additional file 5: GO analysis of target genes of differentially expressed miRNAs in ovaries of *D. melanogaster* induced by *Wolbachia* infection. (XLS 305 kb)

Additional file 6: List of primers used for qRT-PCR or PCR analysis in this work. (XLS 36 kb)

## Data Availability

The datasets generated and/or analyzed during the current study are publicly available at NCBI-SRA (www.ncbi.nlm.nih.gov/sra) accession: SRP136211.

## Abbreviations

CYP: Cytochrome P450
DEGs: Differentially expressed genes
Dmel T: *Drosophila melanogaster* treated with tetracycline (without *Wolbachia*)
Dmel *w*Mel: *Drosophila melanogaster* infected with *w*Mel *Wolbachia*
GO: Gene Ontology
KEGG: Kyoto Encyclopedia of Genes and Genomes
PIWI Interacting RNA: piRNA
RNA-seq: RNA sequencing
sRNA-seq: Small RNA sequencing
